# Clinical Presentation of Inherited Metabolic Diseases in Newborns Hospitalised in an Intensive Care Unit

**DOI:** 10.34763/jmotherandchild.20232701.d-23-00021

**Published:** 2023-10-16

**Authors:** Catarina Teixeira, Catarina Cordeiro, Carla Pinto, Luísa Diogo

**Affiliations:** Pediatric Intensive Care Unit, Pediatric Hospital, Coimbra Hospital and University Center, Coimbra, Portugal; Metabolic Unit, Pediatric Hospital, Coimbra Hospital and University Center, Coimbra, Portugal; European Reference Network for Hereditary Metabolic Disorders

**Keywords:** Inborn errors of metabolism, metabolism, paediatric intensive care unit, hyperlactatemia, coma, newborn

## Abstract

**Background:**

The first clinical manifestations of inherited metabolic diseases occur in the neonatal period in up to half of cases, often with nonspecific symptoms, making their recognition challenging. This study aimed to characterise inherited metabolic disease cases with neonatal presentation requiring admission to the paediatric intensive care unit in a Portuguese reference centre for inherited metabolic diseases.

**Material and methods:**

An observational study with retrospective data collection was performed, including all newborns with an inherited metabolic disease admitted to the pediatric intensive care unit between June 2011 and June 2022. Three ‘pathophysiological’ groups were defined: cases due to small molecules, energy deficiency and complex molecules.

**Results:**

Twenty newborns, with a median age at admission of 7.5 days, were included. Thirteen (65%) were female, sixteen (80%) had a small molecule disorder, and four (20%) had diseases of energy defects. Neurological manifestations were the most common, with most newborns presenting symptomatically in the first week of life. There was no difference between the groups in neurological, cardiac, and hepatic involvement and shock at presentation. A symptom-free interval was more frequent in patients with small molecule disorders than the others (p=0.01). The main metabolic changes found were altered plasma amino acids (n=13) and organic aciduria (n=10), creatine kinase elevation (n=13), hyperlactatemia (n=12), metabolic acidosis with increased anion gap (n=8) and hyperammonaemia (n=7). Newborn screening of metabolites helped make a diagnosis in 60% of cases.

Five newborns died due to multiorgan failure (n=3) or refractory cardiogenic shock (n=1), and in one, therapeutic efforts were limited due to an adverse neurological prognosis.

**Conclusion:**

Although the symptoms and signs are often nonspecific, we should suspect inherited metabolic disease when a newborn presents with neurological symptoms after a symptom-free period, however short it might be. Newborns with suspected inherited metabolic disease should be evaluated with simple biochemical tests, and newborn screening should be urgently expanded to start specific treatment earlier, reducing mortality and morbidity.

## Introduction

Inherited metabolic diseases (IMD), also referred to as inborn errors of metabolism, are a broad and heterogeneous group of monogenic diseases. They are defined as ‘any condition in which primary alteration of a biochemical pathway is intrinsic to specific biochemical, clinical and/or pathophysiological features, regardless of whether there are abnormalities in currently available biochemical laboratory tests’ [1]. With the advent of new diagnosis tools, such as next generation DNA sequencing and metabolomics, the number of newly identified IMD is increasing. Currently, the number of IMD mentioned in the Inborn Errors of Metabolism Knowledgebase is 1,872 [2].

Although most IMD are individually rare (<1:2000) or ultrarare (<1:50000), their cumulative incidence is thought to be high but is unknown. Waters et al. estimated the prevalence of IMD worldwide based on 49 published series between 1980 and 2017 at 50.9 per 100,000 live births [3]. In Portugal, the birth prevalence of IMD included in the expanded newborn screening program was estimated as 41.74 per 100,000 live births [4].

The IMD [5] international classification has been simplified in line with Saudubray's clinical classifications: IMD are divided in to three pathophysiological groups, which are helpful with patient diagnostic and therapeutic orientation [6,7,8]. The small molecule IMD group is due to the accumulation/intoxication and deficiency of small molecules caused by an enzyme deficiency. In the group caused by toxic accumulation, acute and/or chronic progressive intoxication occurs. There may be a variable symptom-free interval after birth, and clinical manifestations depend on food composition and/or intercurrent situations associated with catabolism. Amino acid catabolism defects (e.g. maple syrup urine disease, tyrosinemia, methylmalonic acidemia), urea cycle disorders, and sugar intolerances (e.g. galactosemia, fructosemia) are some diseases included in this group [6,8,9].

The second group includes defects of energy production/utilisation, with primary involvement of the liver, muscle, and brain due to defects in membrane transporters or mitochondrial (e.g. fatty acid, respiratory chain) and/or cytoplasmatic defects [6,8,9].

The third group is caused by complex molecule accumulation, deficiency, cell processing, or trafficking. Conditions in this group, which includes lysosomal diseases and congenital glycosylation diseases, are usually permanent and progressive and are unrelated to diet or intercurrences [6,8,9]. IMD can present at any age with any symptoms. They often present acutely with life-threatening conditions requiring immediate intervention [10]. In up to half of cases, the first clinical manifestations occur in the neonatal period [9]. Newborns often present with nonspecific symptoms, common to several other situations, and therefore differential diagnosis is fundamental [7,9]. IMD should be considered in all situations suggestive of sepsis or perinatal asphyxia, especially when the progression is severe or unexpected [7,9]. Common symptoms and signs in the neonatal period are vomiting, refusal to eat, lethargy, coma, hypotonia, convulsions, visceromegaly, and liver or heart failure. In the diagnostic evaluation of these patients, a detailed clinical history, including family history (comprising neonatal deaths, diseases that occur during childhood and consanguinity) is essential [7,9]. Laboratory clues such as hypoketotic hypoglycemia, lactic acidosis, metabolic acidosis, ketosis and/or hyperammonemia should raise the suspicion of IMD [11]. A metabolic newborn screening program should be urgently applied to any sick newborn independent of their age and fed/fasting state since it could disclose a treatable disorder.

Treatment of neonatal IMD should be early and aggressive. In addition to supportive treatment, therapy involves eliminating the intake of precursors of toxic metabolites as well as the administration of calories to promote anabolism [1,9]. Even in countries with ‘expanded’ metabolic newborn screening, IMD remain one of the most frequent causes of treatable intellectual disability [10,12]. It is, therefore, mandatory to include IMD in the differential diagnosis of critically ill newborns to improve their prognosis.

This study aimed to characterise cases of IMD with neonatal presentation requiring admission to the paediatric intensive care unit (PICU) at a Portuguese reference centre for inherited metabolic disorders.

## Material and methods

An observational study with retrospective data collection was performed.

Eligibility criteria were newborns (aged between 0 and 28 days) with a diagnosis of IMD admitted to the PICU of our hospital between June 2011 and June 2022 (11 years).

Data collection was obtained by consulting computer records from the PICU and general hospital databases (B-ICU Care®; SClinico®). The variables analysed were: age (in days), sex, diagnosis, IMD classification, complications of pregnancy, the need for neonatal resuscitation, consanguinity, family history, symptoms, the symptom-free interval, metabolic newborn screening results, changes in physical examinations, laboratory analytics performed at admission (blood count, ammonia, lactate, glycemia, ketonemia, urea, creatinine, calcium, creatine kinase, total cholesterol, triglycerides, uric acid, blood gases, organic acids in urine and plasma, plasma and cerebrospinal fluid amino acids, urine) 2,4-dinitrophenylhydrazine (DNPH) test, genetic assessment, specific treatment for IMD, PICU supportive therapies (mechanical ventilation, cardiovascular support, renal replacement techniques), death, and follow-ups. Postmortem studies, including autopsies, were also evaluated.

Term newborns were defined as those with a gestational age (GA) ≥38 weeks. Adequacy of birth weight (BW) to GA was assessed by Fenton curves, considering as adequate weight for gestational age (AGA) those with a BW between the 10^th^ percentile (P10) and the 90^th^ percentile (P90); small for gestational age (SGA) was defined as those with BW below P10, and large for gestational age (LGA) as those with a BW above P90. Intrauterine growth restriction (IUGR) was considered when the newborn had a rate of foetal growth less than normal in light of the growth potential [13].

Encephalopathy was considered when there were seizures or a reduced level of consciousness either accompanied or not accompanied by decreased tonus and osteotendinous areflexia [14].

For the definition of acute liver failure, the Pediatric Acute Liver Failure Study Group diagnostic criteria were used [15]. Given the age of the patients, all liver failure was considered acute.

For analysis, the cohort was divided into two groups, those with small molecule IMD and others (energy defect diseases). Statistical analysis was performed with the Statistical Package for the Social Sciences®, version 28. Nominal variables were expressed as numbers and percentages. Depending on their distributions, numeric variables were reported as mean and standard deviation (SD) or median and interquartile range (IQR; P25 to P75).

According to Cochran rules, the χ^2^ test or Fisher's exact test was used to compare nominal variables. Regarding quantitative variables, comparisons between groups were made using the parametric independent samples *t*-test and the non-parametric Mann-Whitney U test, as appropriate. The threshold for significance was defined as *p* < 0.05.

## Results

This study included 20 newborns admitted to the PICU, with a median age at the admission of 7.5 days (3.25–9), with 65% (n=13) being female. They belonged to 19 different families and included a pair of twins. Most (n=14; 70%) were full-term newborns. Seventeen (85%) had an appropriate weight for GA, and three (15%) were SGA. All pregnancies had been monitored except one. One foetus had IUGR. There was consanguinity in four families and a history of IMD in the other four. Demographic and clinical data of the newborn cohort are shown in Table 1.

**Table 1.** Newborns’ demographic and clinical data (n=20).

**Variable**	
Age at PICU admission, median (IQR), days	7.5 (3.25–9)
Female gender n (%)	13 (65)
Gestational age, median (IQR), weeks	38.5 (36–39)
Birth weight, mean (SD), grams	2831.3 (523.1)
Neonatal resuscitation n (%)	1 (5)
Pregnancy complications n (%)	6 (30)
Family history of IMD n (%)	4 (20)
Age at onset of symptoms, median (IQR), days	5 (1–7)
Symptom-free interval n (%)	16 (80)
Length of the symptom-free interval, median (OQR), days	5 (1–7)

In the cohort, it was possible to identify 13 different IMD, detailed in Table 2. The most frequent group was that of small molecule disorders (n=16; 80%), intoxication (n=15; 75%) and deficiency (n=1; 5%), followed by energy defects (n=4; 20%). Maple syrup urine disease, organic acidurias and urea cycle disorders were the prevailing diagnoses.

**Table 2.** IMD diagnosed in hospitalised newborns by pathophysiological group.

**Pathophysiological group**	**IMD**	**n**
**Small molecules (accumulation/intoxication) n=15**	Organic aciduria	**4**
	Propionic aciduria	1
	Methylmalonic aciduria	3
	Maple syrup urine disease	**4**
	Urea cycle disorders	**3**
	Citrullinaemia type 1^*^	2
	Ornithine transcarbamylase deficiency (OTC)	1
	Galactosemia	**3**
	- Classic	2
	- Epimerase deficiency	1
	Nonketotic hyperglycinemia	**1**
**Small molecules (deficiency) n=1**	Asparagine synthetase deficiency	**1**
**Energy defects n=4**	Carnitinepalmitoyltransferase 1 deficiency (CPT1)	**1**
	Carnitine-acylcarnitine translocase deficiency (CACT)	**1**
	Multiple oxidative phosphorylation defect (complexes IV-V-II+IV)	**1**
	Combined oxidative phosphorylation defect type 13	**1**

* The two cases of citrullinaemia type 1 are twins.

There was no significant difference between the two IMD groups in GA (median of 39 weeks in the small molecules group versus 37 weeks in the energy defects group, *p*=0.2) and BW (median of 2955 g in the small molecules group versus 2790 g in the energy defects group, *p* = 0.5).

The most frequent clinical manifestations were encephalopathy (n=17; 85%), hypotonia (n=12; 60%), feeding refusal (n=14; 70%), hypoglycaemia (n=7; 40%) and seizures (n=8; 40%). Five newborns had shock at clinical presentation (four in the small molecules group and one in the energy defects group). There was no significant difference between the two IMD groups concerning neurological manifestations, cardiac and hepatic involvement, and presentation with shock (Table 3).

**Table 3.** Comparison of clinical manifestations between the two IMD groups.

	**Small molecules n=16**	**Energy defects n=4**	***p***
**Symptom-free interval n (%)**	15 (93.8)	1 (25)	0.01^*^
**Length of the symptom-free interval, median (IQR), days**	5.5 (0–7.8)	3 (0–6.8)	0.3^**^
**Encephalopathy n (%)**	14 (87.5)	3 (75)	0.5^*^
**Hypotonia n (%)**	10 (62.5)	2 (50)	0.2^*^
**Seizures n (%)**	6 (37.5)	2 (50)	1^*^
**Acute liver failure n (%)**	3 (18.8)	0	1^*^
**Heart failure n (%)**	2 (12.5)	2 (50)	0.2^*^
**Cardiac structural change n (%)**	3 (18.8)	2 (50)	0.4^*^
**Death n (%)**	4 (25)	1 (25)	0.7^*^

Note: Each newborn may present more than one symptom

* Fisher's exact test;

** Mann-Whitney U test

A symptom-free interval was significantly more frequent in the small molecule group (*p*=0.01) (Table 3). The length of the symptom-free interval was similar between the two groups (Table 3).

The ‘expanded’ metabolic newborn screening of IMD, based on 20 profile mass spectrometry/mass spectrometry (MS/MS), was positive in 60% of patients (n=12): ten had small molecule diseases and two newborns had energy defects. Clinical manifestations were present before metabolic newborn screening results were available in all but one of the twins with citrullinaemia type 1.

The main analytical changes found were altered plasma amino acids (AA) (n=12; 60%) and urine organic acids (OA) (n=10; 50%), creatine kinase elevation (n=13; 65%), hyperlactatemia (n=12; 60%), glomerular renal function impairment (n=11; 55%), metabolic acidosis with increased anion gap (n=8; 40%) and hyperammonemia (n=7; 35%).

Hyperlactatemia was present in all newborns in the energy defects group. In the small molecules group, it was more frequent in patients with galactosemia (classic (n=2) and epimerase deficiency (n=1)), maple syrup urine disease (n=2), and urea cycle defects (n=2).

Hyperammonemia was present in seven patients, most belonging to the small molecules group (n=6), with a diagnosis of urea cycle defects (n=3), classic galactosemia (n=1) and organic aciduria (n=1).

Table 4 describes the main alterations of the diagnostic tests by the pathophysiological disease group.

**Table 4.** Main alterations of diagnostic tests by pathophysiological group of IMD.

**Diagnostic tests**	**Small molecules group (n=16)**	**Energy defects group (n=4)**	*p* ^*^
Altered plasma aminoacids, n (%)	12 (75)	1 (25)	0.2
CK elevation, n (%)	11 (68.8)	2 (50)	0.6
Hyperlactatemia, n (%)	8 (50)	4 (100)	0.4
Glomerular renal dysfunction, n (%)	8 (50)	3 (75)	0.6
Metabolic acidosis with increased anion gap, n (%)	7 (43.8)	1 (25)	0.5
Organic aciduria, n (%)	7 (43.8)	3 (75)	0.2
Abnormal urine aminoacids, n (%)	7 (43.8)	0	0.08
Hyperammonemia, n (%)	6 (37.5)	1 (25)	1
DNPH urine test - positive, n (%)	6 (37.5)	0	0.2
Hypoglycemia, n (%)	6 (37.5)	2 (50)	0.4
Thrombocytopenia, n (%)	5	0	0.2
Leukopenia, n (%)	0	1	0.2

* Fisher's exact test

Legend: CK - Creatine kinase; DNPH - 2,4-dinitrophenylhydrazine

Echocardiograms performed in 11 patients disclosed structural changes in five: coarctation of the aorta in a patient with multiple oxidative phosphorylation defects (complexes IV-V-II+IV); pericardial effusion in a newborn with carnitine-acylcarnitine translocase deficiency; mitral and aortic insufficiency in one case of methylmalonic aciduria; isolated mitral insufficiency in one patient with classic galactosemia and left ventricular hypertrophy in a case with maple syrup urine disease.

An electroencephalogram was performed in nine patients, with anomalies detected in seven, with a predominance of paroxysmal activity (n=5), followed by burst suppression in two newborns (one with asparagine synthetase deficiency and another one with nonketotic hyperglycinemia). Abdominal and pelvic ultrasounds were done in ten patients. Liver enlargement was detected in two (classic galactosemia and ornithine transcarbamylase deficiency [OTC] deficiency).

Genetic studies (by Next-Generation sequencing methods and Sanger sequencing in selected cases) confirmed the diagnosis in all patients except two: the boy (born in 2013 and deceased in 2017) with multiple oxidative phosphorylation defects (muscle biopsy) and the most recent case detected by metabolic newborn screening, a CPT1, still under study. In the patient born in 2021, the diagnosis of combined oxidative phosphorylation defect 13 was performed by Whole Exome sequencing (WES).

Regarding treatment, all received supportive care, with 15 requiring invasive mechanical ventilation, 4 requiring non-invasive mechanical ventilation, and 6 requiring cardiovascular support. All patients with maple syrup disease and those with OTC deficiency underwent continuous venovenous hemodiafiltration (CVVHDF; n=5, 25%). As for specific treatment, all patients were administered specific diets according to their IMD.

Genetic assessment was performed in all newborns, and pathogenic variants were identified in 18 cases. In the newborn with multiple oxidative phosphorylation defects (complexes IV-V-II+IV), the genetic study was negative. Genetic analysis of the most recent case (CPT1 deficiency) is ongoing.

Five of the 20 newborns (25%) died during the neonatal period due to multiorgan failure (n=3), refractory cardiogenic shock (n=1) and limitation of therapeutic effort due to adverse prognosis (n=1). Four had small molecule IMD (methylmalonic aciduria, OTC deficiency, classic and epimerase deficiency galactosemia), and one was in the energy defects group (carnitine-acylcarnitine translocase deficiency). There was no statistically significant mortality difference in the neonatal period between the diagnosis groups (Table 3). One of the patients underwent a skin and muscle biopsy, allowing the final diagnosis of multiple oxidative phosphorylation defect (complexes IV-V-II+IV).

In postmortem studies, various tissue analysis was performed involving skin (n=5), liver (n=2) and skeletal and cardiac muscle (n=2). They contributed to the final diagnosis in two patients, the only who underwent autopsies: classic and epimerase deficiency galactosemia. There were two deaths during follow-up, both at two years of age, in patients with small molecule defects and severe neurologic impairment: nonketotic hyperglycinemia and asparagine synthetase deficiency.

Three of the 13 (23%) surviving patients underwent liver transplantation, two with a diagnosis of maple syrup urine disease (both at two years of age) and one with propionic aciduria (at seven years of age).

Five (38%) patients had global psychomotor development retardation/intellectual deficiency.

## Discussion

The neonatal period is characterised by intense catabolism, so the presentation of IMD frequently occurs in this period of life. Cases may present with life-threatening episodes requiring immediate intervention and admission to neonatal ICU/PICU [10]. In this study, over 11 years, 20 newborns with IMD required intensive care. There was a higher prevalence of IMD in females, different from what is described in the literature. There seems to be a higher prevalence of autosomal recessive inherited IMD in males [16].

The most frequent pathophysiological group of IMD in our patients was small molecule diseases, with organic acidurias and maple syrup urine disease standing out, which agrees with other publications [17,18].

There was consanguinity in 20% (4 of 20) of the families in our cohort, a value lower than the 34%–87% described by other authors [14]. A family history of IMD was observed in 20%, although in different cases. Consanguinity and a family history of IMD should lead to the suspicion of IMD. However, their absence should not exclude the hypothesis of an IMD, as in 60% of our cohort there was neither consanguinity nor previous IMD in the family [7,10].

Many IMD have a severe neonatal presentation and are a primary cause of death in newborns and infants [12,13]. The newborn has a limited repertoire of responses to severe illness, so IMD may present with nonspecific symptoms that can easily be attributable to other more common causes, such as sepsis [7]. It should be noted that a sepsis diagnosis does not necessarily rule out the presence of an IMD. Infection may cause the decompensation of an underlying IMD. Thus, a high level of care is required to reach a correct diagnosis. Early recognition of these conditions is essential to initiate targeted intervention in treatable situations, with implications for prognosis [19,20].

In this cohort, most newborns (70%) had symptoms in the first week of life, and only one presented during the third week of life (at 19 days).

The newborns in the small molecule IMD group typically had a symptom-free period after birth. These are usually full-term infants who, after a period during which intoxication (with specific amino acids or galactose) starting after birth is progressive, deteriorate rapidly for no apparent reason and do not respond to symptomatic therapy [7,8]. This clinical course contrasts with other groups, in which the clinical presentation has more variable severity and often had no symptom-free period [7]. Long-chain fatty acid oxidation disorders are an exception, which are associated with energy deficiency and intoxication and can have a similar clinical presentation [7].

Neurologic involvement with encephalopathy, hypotonia and seizures were our population's most frequent symptoms and signs, which agrees with other studies, since 85% of IMD display predominant neurological manifestations [21].

Encephalopathy in IMD usually results from accumulation in the brain of a small diffusible metabolite or precursor (like ammonia or branched-chain alfa-ketoacids), a deficiency of an essential product (like glucose) or a defective transport process (like a carnitine transporter). Most of these metabolites cross the placenta and are cleared, which may explain why affected newborns are usually normal at birth [10].

Regardless of IMD type, many patients present with neurologic symptoms that may be secondary to elevated ammonia levels, metabolic acidosis, hypoglycemia, or no immediately identifiable metabolite abnormality [22].

The rate of neurologic deterioration varies considerably depending on the nature and severity of the metabolic defect. Some newborns may have only a very short phase when they appear well, and it can be difficult to distinguish IMD from birth asphyxia. Such as occurs with sepsis, birth asphyxia can be a clinical manifestation of an IMD and aggravate its prognosis. Others may appear well for several days and deteriorate more gradually, as in our sample [22].

Thus, we should suspect IMD when a newborn presents with neurological symptoms after a symptom-free period, however short it might be.

Although isolated neonatal seizures are not suggestive of IMD, they can be the first manifestation before neurologic deterioration ensues. Burst suppression patterns, although not pathognomonic, should raise the suspicion of an IMD, namely of nonketotic hyperglycinemia, which happened in our case. Awareness is important since it allows a prompt diagnostic work-up and adequate treatment, reducing neurological impairment [21].

In our cohort, four newborns presented with heart failure and five with shock. Primary cardiac involvement in IMD is rare, being more frequent in cases with energy defects, namely in fatty acid oxidation disorders. Arrhythmias or conduction abnormalities may be present, as well as cardiomyopathy and pericardial effusion [22].

Secondary cardiac involvement with global cardiac dysfunction caused by acidosis and metabolic stress should be considered to optimise treatment [22].

The Portuguese newborn screening program, a national coverage population program, including about 100% of all newborns, has been available in Portugal since 2005 [23]. It is an ‘expanded’ program, based on amino acids and acylcarnitines profile performed by MS/MS and includes 24 IMD (selected aminoacidopathies, organic acidurias and mitochondrial fatty acid β-oxidation disorders). It is an essential diagnostic tool for any sick newborn and should be performed as soon as possible, independently of recommended age and fed state. In fact, the catabolic state associated with the disease enhances the probability of metabolite detection [24]. In this cohort, screening helped in enabling the diagnosis of IMD in 60% of cases. Although all (four) cases of maple syrup urine disease presented before the screening result was known, it was decisive for emergent treatment decisions.

With the advent of next-generation DNA sequencing techniques, progress in diagnosing IMD has been exponential. In every sick newborn, even if an infectious process is detected, metabolic newborn screening result should be immediately obtained. If it is negative, and clinical and routine analysis do not orientate for a metabolic cause (like in galactosemia), and no other diagnosis is reached, WES should be considered [25], as occurred in the patient of our cohort with the diagnosis of combined oxidative phosphorylation defect 13. Molecular diagnosis is essential for genetic counselling of families, allowing the prenatal diagnosis in subsequent pregnancies. However, even if it confirms the diagnosis, its availability is not immediate [7]. Thus, the guidance for these patients in a NICU/PICU setting must rely on clues from the clinical history, clinical manifestations and the results of laboratory tests [25]. The initial evaluation of a sick newborn should include blood count, arterial blood gases and plasma glucose, ions, urea, creatinine, aminotransferases, creatine kinase, uric acid, lactate, ammonia, amino acids, capillary ketonemia, urine ketones and organic acids (including orotic acid if hyperammonemia is present). Although not specific, a urine DNPH test for alpha-ketoacids is a rapid bedside test that may reinforce the suspicion of maple syrup urine disease and encourage the implementation of emergent anabolic-promoting measures before branched-chain amino acid levels are known [11]. Our results support these guidelines since most newborns presented alterations in these diagnostic tests.

A sick newborn may present with mild metabolic acidosis. An underlying small molecule (accumulation/intoxication) IMD should be suspected in severe metabolic acidosis and increased anion gap. Concomitant metabolic changes such as hypoglycemia, ketosis, hyperlactatemia, and hyperammonemia may be helpful in diagnosis [11]. Most IMD that present with overwhelming metabolic acidosis and ketosis are organic acidemias (i.e. methylmalonic acidemia, propionic acidemia, isovaleric acidemia). Metabolic acidosis with hypoglycemia and no ketosis may suggest mitochondrial fatty acid oxidation defects [11].

Hyperlactatemia is most often due to shock or hypoxemia. Once a ‘non-metabolic’ cause is excluded, mitochondrial energy disruption by toxic metabolites like fatty acid oxidation disorders, organic acidurias and, rarely, urea cycle defects should be suspected. Other inherited causes of persistent hyperlactatemia include disorders of glycogen metabolism and gluconeogenesis and disorders directly affecting the Krebs cycle or pyruvic acid metabolism [11]. In our cases, hyperlactatemia was present in all newborns with energy defect IMD and in two with urea cycle defects.

Ammonia assessment is another first-line test that should be performed in all newborns presenting with neurological deterioration, along with sepsis screening. Elevated ammonia should raise the suspicion of IMD and require rapid intervention to prevent brain damage [22]. In our cohort, an ammonia increase was more frequent in patients with small molecule defects (n=6) and in those with a diagnosis of urea cycle defects (n=3), classic galactosemia (n=1), and organic aciduria (n=1).

The initial therapeutic approach for patients with small molecule accumulation includes the promotion of anabolism with high caloric intake and insulin intravenous perfusion and, in selected cases, the mechanical removal of toxic metabolites and supplementation with enzymes or cofactors, making up for the lack of a metabolite deficiency [26]. Most of our patients in the small molecule group were managed with diet, supplementation with enzymes or cofactors and mechanical removal of toxic metabolites. These were the principles used in treating the 20 newborns included in this study.

Five patients underwent renal replacement therapies, with CVVHDF being the most used renal replacement technique during the PICU stay (25%). As described in the literature, this is the most efficient technique for reducing toxic metabolites and, consequently, in reducing neurological sequelae [10,27, 28].

Mortality was high (7 of 20 patients; 35%) in our series. All these patients died before two years of age, and the current median of age of survivors is four years (2–10). Five (25%) newborns admitted to the PICU died during the neonatal period, with no significant difference between pathophysiology groups. This high mortality trend in newborns with IMD has been described in other studies. Most die during the first episode of disease decompensation, even before diagnosis [3,16,26]. Nonspecific symptoms contribute to the delay in diagnosis and the initiation of specific management in potentially treatable situations, with progression to multiorgan failure and death [3]. For most IMD, early presentation is associated with high disease severity. Some newborns die even with early aggressive treatment [26]. On the other hand, not all IMD are amenable to specific treatments, contributing to the high mortality rate. In one patient with asparagine synthase deficiency, symptomatic treatment was withdrawn due to an adverse neurologic prognosis.

There has been a gradual increase in the awareness of IMD over time, and earlier diagnoses may decrease mortality. In this context, the development of mechanical removal of toxic metabolites techniques and progress in liver transplantation are most relevant, as these have increased the survival and quality of life of some of these patients.

Our study has some limitations, namely the small sample size and the lack of an equal number of patients in each of the pathophysiological groups of IMD, making it difficult to draw statistically valid conclusions from the results and limiting their extrapolation to the remaining population. Its retrospective nature is another limitation, increasing the possibility of bias and missing data. Despite these constraints, this study has been essential for our clinical practice since it allowed us to reflect on our procedures and optimise them.

### Key Points

- IMD may present early in the neonatal period with nonspecific clinical manifestations and a high mortality rate.
- IMD should be suspected and considered in the differential diagnosis of any sick newborn since that can be severe and require admission to PICU once renal replacement therapies and other supportive therapies may be needed to improve the prognosis.
- Sick newborns should be evaluated for IMD, with urgent ‘expanded’ newborn screening, to start specific treatment as early as possible and reduce mortality and morbidity.
